# Association between occurrence time of myocardial injury after noncardiac surgery and long-term functional capacity: a secondary analysis of a prospective study

**DOI:** 10.1080/07853890.2025.2552936

**Published:** 2025-09-02

**Authors:** Tian-Ying Tang, Jia-Ming Liu, Hua-Min Liu, Jing Zhang, Fu Zhang, Bing-Cheng Zhao, Ke-Xuan Liu, for the PREVENGE-CB Collaborators

**Affiliations:** a Department of Anaesthesiology, Nanfang Hospital, Southern Medical University; The key Laboratory of Precision Anaesthesia & perioperative Organ Protection, Guangzhou, Guangdong, China; b School of Nursing, Southern Medical University, Guangzhou, China

**Keywords:** Disability, long-term functional capacity, myocardial injury, noncardiac surgery, troponin T surveillance

## Abstract

**Background:**

Myocardial injury after noncardiac surgery (MINS) is associated with poor prognosis. The effect of different occurrence time of MINS on long-term functional capacity remains unclear in population with high cardiovascular risk.

**Patients and methods:**

This cohort study included adult patients with increased cardiovascular risk undergoing elective major noncardiac surgery from June 2019 to September 2021. Patients with MINS were stratified in two groups on the basis of the occurrence time of MINS: within 24  hour (h) or after 24 h. The primary endpoint was disability at 180 days after surgery, evaluated by World Health Organization Disability Assessment Schedule 2.0. Disability was defined as ≥25% or an increase of 8%. Multivariable logistic regression was adopted to explore the association between occurrence time of MINS and primary endpoint. Propensity score weighting, including inverse probability weighting and overlap weighting, and subgroup analysis were used to explore the relationship further.

**Results:**

2469 participants were included, of which 178 (7.2%) participants developed MINS within 24 h and 83 (3.4%) after 24 h. A total of 378 (15.3%) participants developed disability at 180 days after surgery, with an unweighted odds ratio (OR) of 1.97 (95% confidence intervals [CIs]: 1.17–3.32) for patients who suffered MINS after 24 h and weighted OR of 2.25 (95%CIs: 1.10–4.63) and 2.11 (95%CIs: 1.23–3.63) by IPW and OW, respectively. Findings were conserved in the subgroup analysis.

**Conclusion:**

MINS occurring after 24 h was associated with worsen long-term functional capacity after surgery, whereas MINS occurring within 24 h was not.

## Introduction

Over 230 million noncardiac surgeries are performed in the world every year, and this number is still increasing [1]. One in 6 patients experienced a complication, and 1 in 35 patients who suffered a complication subsequently died in the index hospitalisation; mortality was high (7.5%) within 1 year after surgery [2]. Upon discharge, patients with or without complications pay more attention to returning to their preoperative lifestyle or functional capacity rather than traditional research endpoints, such as mortality and morbidity, which clinicians understandably focus on [3,4]. Postoperative complications evaluation during hospitalisation and functional capacity follow-up after discharge are important aspects of postoperative recovery, but consideration of the effect of postoperative complications on overall functional capacity is inadequate.

Myocardial injury after noncardiac surgery (MINS), a silent postoperative complication characterised with troponin elevation and absence of ischaemic features, was independently associated with increased 30-day mortality [5]. Therefore, guidelines recommend single troponin measurement before surgery and a series 48–72 h postoperatively for high-risk population [6–8]. For patients with mild preoperative troponin elevations, higher troponin level was associated with higher 30-day mortality after surgery, and longer time to surgery appeared to reduce this risk [9]. After surgery, elevated troponin within 24 h was associated with the development of noncardiac organ dysfunction during hospitalisation [10]; however, it is unclear that whether the prognosis improve or not along with time after surgery. Hence, this study focuses on the relationship between the occurrence time of MINS and long-term functional capacity.

## Patients and methods

### Study design and population

The research was based on the data collected from PREdiction of Vascular Events after major Noncardiac surGEry with preoperative Cardiac Biomarkers study (PREVENGE-CB), a prospective cohort which aimed to assess the ability of preoperative N-terminal pro-B-type natriuretic peptide and troponin T to predict postoperative adverse cardiovascular events. The study was approved by the Ethical Committee of Southern Medical University Nanfang Hospital (Guangzhou, China; No. NFEC-2019-083; approved on May 8, 2019) and registered at the Chinese Clinical Trial Registry (ChiCTR1900023779). The Strengthening the Reporting of Observational Studies in Epidemiology reporting guidelines for cohort studies was attached in supplementary material (Supplementary Table S1). Briefly, we included patients aged ≥45 years who had at least one known cardiovascular diseases (coronary heart disease, chronic heart failure, stroke or peripheral vascular disease) or carried two or more risk factors for cardiovascular diseases from June 2019 to September 2021. Venous blood samples were collected at the time between recruitment and anaesthesia induction and on the morning of the first and second days after surgery to measure troponin T concentrations via the fifth-generation elecsys troponin T assay (Roche Diagnostics, China), with a 99th percentile URL of 14 ng/L for both men and women. Additional troponin T measurements were performed as needed during the hospitalisation. All the participants provided written informed consent. Participants without troponin T measurement before or after surgery, who missed telephone follow-up at 180 days postoperatively or died in the index hospitalisation were excluded from this analysis. Detailed inclusion and exclusion criteria have been indicated in our previous study [11].

### Data collection and definition

Clinical data, including demographic characteristics, comorbidities, examinations, functional capacity, surgery information, type of anaesthesia and postoperative complications, were collected through interview or from electronic medical records. Cardiovascular comorbidities involve coronary heart disease, chronic heart failure, stroke, peripheral vascular disease, hypertension and atrial fibrillation. Postoperative cardiovascular complications include myocardial infarction, cardiac arrest, heart failure, stroke, new atrial fibrillation and pulmonary embolism. MINS is defined as a postoperative troponin T level ranging from 20 ng/L to less than 65 ng/L with an absolute change of at least 5 ng/L or a postoperative troponin T level of at least 65 ng/L ascribed to myocardial ischaemia, without ischaemic features, following the criterion of the Vascular Events in Noncardiac Surgery Patients Cohort Evaluation (VISION) study [5]. Disability was evaluated through the 12-item World Health Organization Disability Assessment Schedule 2.0 (WHODAS 2.0) face to face at 1 day before surgery and by telephone-based version at 180 days after surgery. The 12-item WHODAS 2.0 was recommended by the Standardised Endpoints in Perioperative Medicine-Core Outcome Measures in Perioperative and Anaesthetic Care (StEP-COMPAC) working group as functional capacity measurement, in which the validity and reliability of the telephone-based version in long-term outcome were assessed in diverse surgical cohort [12–14]. For patients with a preoperative disability score of <25%, disability is defined as a disability score ≥25% at follow-up. If the preoperative disability score is ≥25%, an increase of 8% after surgery denotes a disability [14]. Death is maximal disability. Definitions or diagnosis criteria of comorbidities and postoperative complications are listed in supplementary material.

### Statistical analysis

Continuous variables were described as mean (standard deviation) or median (interquartile range) and compared by Student’s *t* test or Mann–Whitney *U* test or Kruskal–Wallis *H* test or one-way analysis of variance, as appropriate. Categorical variables were presented as count (percentage) and compared via Pearson χ^2^ test or corrected χ^2^ test. Variance inflation factors (VIFs) were calculated to assess the multicollinearity of all variables in the model. Multivariable logistic regression analyses were performed to explore the association between occurrence time of MINS and disability at 180 days after surgery. Several models were applied: the first model only included the occurrence time of MINS; the second model contained preoperative variables, surgery information, type of anaesthesia and occurrence time of MINS; the third model adjusted all confounders, except cardiovascular complications; and complete analysis was performed finally. Subgroup analysis was conducted in four special groups: participants who underwent cancer surgery, participants except orthopaedic and spine surgery, participants without cardiovascular complications and participants without any complications other than MINS.

Propensity score weighting (PSW) methods were adopted as sensitivity analysis. A multivariable logistic regression model including all covariates was utilised to calculate the propensity score for inverse probability weighting (IPW) and overlap weighting (OW). Briefly, a weight calculated from multivariable logistic regression was assigned to each subject, resulting in a pseudo-population where baseline characteristics are independent of exposure status. Standardised mean difference (SMD) indicates the difference between varying groups, and effect size indices of 0.2, 0.5 and 0.8 can be used to represent small, medium and large effect sizes, respectively [15]. All hypothesis tests were two-tailed with a *P*-value of 0.05 or smaller as significant. The statistical analysis was conducted using IBM SPSS Statistics for Windows, Version 25.0 (IBM Corp., Armonk, NY, USA) and R (version 4.4.1, R Foundation for Statistical Computing, Vienna, Austria).

## Results

### Study population characteristics

**Figure 1.**
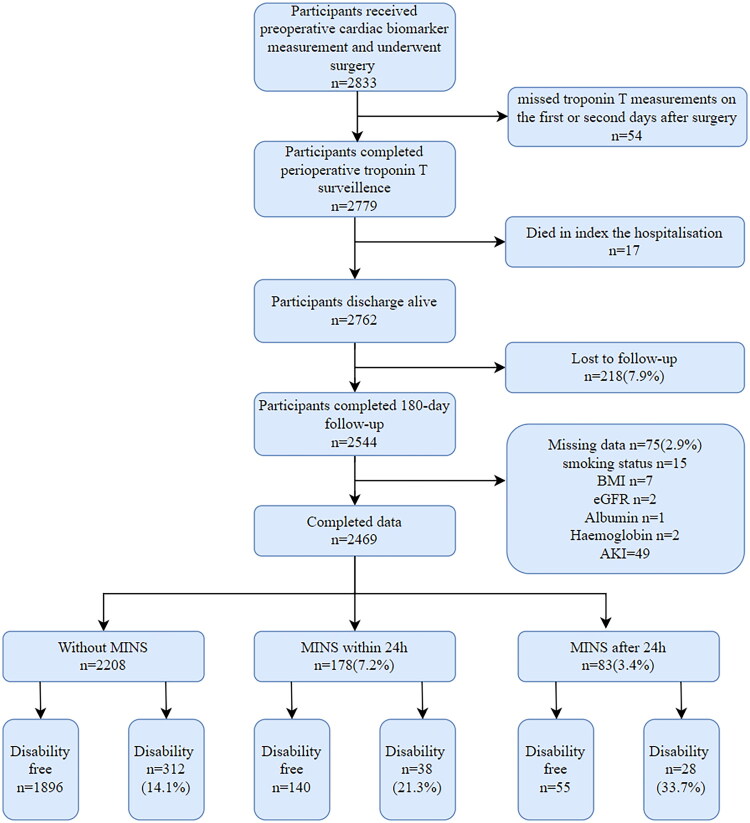
Flowchart of the study.

2469 participants were included in the study population (Figure 1), with a median age of 68 years, 60.4% male, 65.8% had known cardiovascular comorbidity, 30.7% known diabetes mellitus and 59.4% known cancer and 26.5% participants received orthopaedic or spine surgery. One hundred seventy-eight (7.2%) participants developed MINS within 24 h after surgery and 83 (3.4%) after 24 h (Supplementary Table S2). The 378 (15.3%) participants who suffered disability at 180 days after surgery were older, smoked less often, were more often diagnosed with cancer and were characterised by lower preoperative levels of albumin and haemoglobin. Patients who experienced MINS, regardless of whether within or after 24 h, were those who suffered AKI, infection, pulmonary complications or major bleeding more during hospitalisation (Supplementary Table S2). Detailed characteristics are provided in Table 1.

**Table 1.** Characteristics of participants stratified by disability.

	All *n* = 2469	Without disability *n* = 2091 (84.7%)	With disability *n* = 378(15.3%)	*P*-value	SMD
Age (years)	68.0 (63.0,74.0)	68.0 (62.0,73.0)	71.0 (66.0,77.0)	<0.001	0.432
Female	977(39.5)	835 (39.9)	142 (37.6)	0.419	0.049
BMI, kg/m^2^	23.1 (20.8,25.5)	23.3 (21.0, 25.7)	21.9 (19.5, 24.6)	<0.001	0.339
Smoking status				0.029	0.154
Never	1562 (63.3)	1308 (62.6)	254 (67.2)		
Former	270 (10.9)	223 (10.7)	47 (12.4)		
Current	637 (25.8)	560 (26.8)	77 (20.4)		
Cardiovascular comorbidities	1624 (65.8)	1392 (66.6)	232 (61.4)	0.057	0.108
Chronic obstructive pulmonary disease	86 (3.5)	68 (3.3)	18 (4.8)	0.187	0.077
Diabetes mellitus				0.044	0.145
No	1713 (69.4)	1431 (68.4)	282 (74.6)		
Diabetes mellitus on oral drug	525 (21.3)	455 (21.8)	70 (18.5)		
Diabetes mellitus on insulin	231 (9.4)	205 (9.8)	26 (6.9)		
Liver	607 (24.6)	515 (24.6)	92 (24.3)	0.955	0.007
Cancer	1467 (59.4)	1209 (57.8)	258 (68.3)	<0.001	0.217
eGFR, mL/min	84.4 (69.5,92.7)	84.7 (69.8, 92.9)	82.3 (67.62, 90.9)	0.016	0.128
Albumin, g/L	38.0 (35.0,40.6)	38.3 (35.4, 40.8)	36.3 (33.5, 38.8)	<0.001	0.434
Haemoglobin, g/L	123.0 (107.0,135.0)	124.0 (109.0, 136.0)	113.0 (97.0, 129.0)	<0.001	0.423
Minimally invasive surgery	1470 (59.5)	1295 (61.9)	175 (46.3)	<0.001	0.318
Blood transfusion during surgery	275 (11.1)	205 (9.8)	70 (18.5)	<0.001	0.252
Type of surgery				<0.001	0.410
Foregut or hepatopancreatobiliary	566 (22.9)	461 (22.0)	105 (27.8)		
Intestinal	480 (19.4)	394 (18.8)	86 (22.8)		
Orthopaedic	434 (17.6)	353 (16.9)	81 (21.4)		
Thoracic(nonoesophageal)	371 (15.0)	340 (16.3)	31 (8.2)		
Urological	250 (10.1)	219 (10.5)	31 (8.2)		
Spine	219 (8.9)	206 (9.9)	13 (3.4)		
Other^a^	149 (6.0)	118 (5.6)	31 (8.2)		
Type of anaesthesia				0.002	0.185
Regional anaesthesia alone	269 (10.9)	208 (9.9)	61 (16.1)		
General plus regional anaesthesia	54 (2.2)	47 (2.2)	7 (1.9)		
General anaesthesia alone	2146 (86.9)	1836 (87.8)	310 (82.0)		
MINS				<0.001	0.260
No	2208 (89.4)	1896 (90.7)	312 (82.5)		
Within 24 h	178 (7.2)	140 (6.7)	38 (10.1)		
After 24 h	83 (3.4)	55 (2.6)	28 (7.4)		
Cardiovascular complications	98 (4.0)	60 (2.9)	38 (10.1)	<0.001	0.295
AKI	180 (7.3)	140 (6.7)	40 (10.6)	0.01	0.139
Infection	203 (8.2)	143 (6.8)	60 (15.9)	<0.001	0.288
Pulmonary complications	18 (0.7)	9 (0.4)	9 (2.4)	<0.001	0.166
Intestinal complications	69 (2.8)	48 (2.3)	21 (5.6)	0.001	0.168
Major bleeding	81 (3.3)	56 (2.7)	25 (6.6)	<0.001	0.188

Values in parentheses are percentages or interquartile ranges.

^a^Included gynaecologic (81, 54.3%), head and neck (47, 31.5%) and vascular (21,14.1%) surgeries.

### Association between occurrence time of MINS and disability at 180 days after surgery

The results of multivariable logistic regression models for the association between the occurrence time of MINS and disability risk are shown in Table 2. All VIFs were less than two (Supplementary Table S3), indicating that no multicollinearity existed among the studied variables. In model 1, in which any other confounders were unadjusted, MINS occurring within 24 h after surgery was positively associated with disability at 180 days after surgery (odds ratio [OR]: 1.65, 95% confidence intervals [CIs]: 1.13–2.40, *P* = 0.009), the effect size decreased gradually as more information was considered in subsequent models; As a contrast, MINS occurring after 24 h was positively associated with outcome in all models, whose effect lessened by degrees with covariable regulation. Interestingly, similar effects were observed in models 3 (OR: 2.09, 95%CIs: 1.25–3.48, *P* = 0.005) and 4 (OR: 1.97, 95%CIs: 1.17–3.32, *P* = 0.01), that is, the effect of MINS occurring after 24 h was stable whether postoperative cardiovascular complications were considered or not.

**Table 2.** Association between occurrence time of MINS and disability at 180 days based on logistic regression.

	Model 1	Model 2	Model 3	Model 4
MINS				
No	Ref	Ref	Ref	Ref
Within 24h, OR (95% CI), *P*	1.65 (1.13,2.40), 0.009	1.16 (0.77, 1.74), 0.481	1.06(0.70,1.60), 0.785	0.97(0.63,1.47), 0.865
After 24h, OR (95% CI), *P*	3.09 (1.93,4.95), <0.001	2.23 (1.34,3.71), 0.002	2.089(1.25,3.48), 0.005	1.97(1.17,3.32), 0.010

### Association between occurrence time of MINS and disability at 180 days after surgery using PSW regression

Maximal SMD of each baseline before and after PSW as shown in Table S2. Participants with MINS 24 h after surgery were more likely older, smoked less often and had lower levels of albumin and haemoglobin before surgery. Participants who developed MINS, whether within or after 24 h, seemed to experience higher risk of other complications (Table S2). The results of multivariate logistic regression analysis based on pseudo-population generated by IPW or OW mirrored the results of unweighted analysis (Tables 3 and 4), the 95%CIs of effect size for MINS within 24 h crossed 1 in all the model.

**Table 3.** Association between occurrence time of MINS and disability at 180 days after surgery using propensity score IPW regression.

	Model 1	Model 2	Model 3	Model 4
MINS				
No	Ref	Ref	Ref	Ref
Within 24 h, OR (95% CI), *P*	0.70 (0.43,1.33), 0.145	0.66 (0.39,1.10), 0.111	0.64 (0.38,1.10), 0.089	0.64 (0.38,1.10), 0.094
After 24 h, OR (95% CI), *P*	1.92 (0.98,3.79), 0.059	2.13 (1.04,4.36), 0.038	2.24 (1.09,4.59), 0.028	2.25 (1.10,4.63), 0.027

**Table 4.** Association between occurrence time of MINS and disability at 180 days after surgery using propensity score OW regression.

	Model 1	Model 2	Model 3	Model 4
MINS				
No	Ref	Ref	Ref	Ref
Within 24 h, OR (95% CI), *P*	0.96 (0.62,1.52), 0.888	0.91 (0.55,1.49), 0.705	0.91 (0.54,1.51), 0.0.70846	0.88 (0.53,1.49), 0.652
After 24 h, OR (95% CI), *P*	1.80 (1.07,3.01), 0.026	2.02 (1.17,3.48), 0.011	2.13 (1.24,3.65), 0.00609	2.11 (1.23,3.63), 0.007

### Subgroup analysis

**Figure 2.**
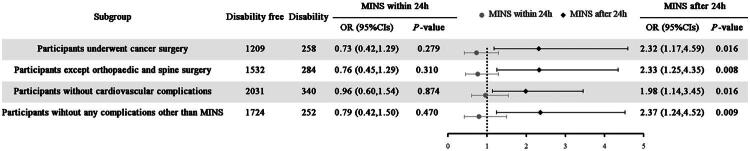
Subgroup analysis of the association between occurrence time of MINS and disability at 180 days after surgery.

Subgroup analysis, conducted on all covariates using a fully adjusted logistic regression model, shared similar results with the models above. As shown in Figure 2, in the all four models, the OR for MINS after 24 h consistently demonstrated an effect size around 2, whereas the 95%CIs for MINS within 24 h crossed 1.

## Discussion

This prospective cohort study of patients with cardiovascular risk investigated the relationship between different occurrence time of MINS and disability at 180 days after major noncardiac surgery. In our analysis, patients with MINS were stratified in two groups on the basis of the occurrence time of MINS, namely, within 24 h or after 24 h, referring to the elimination rate for troponin T in NTEMI patients [16]. This study revealed that MINS occurring after 24 h was associated with deteriorated long-term functional capacity, regardless of the cases with or without postoperative cardiovascular complications.

Participants who underwent cancer surgery and orthopaedic or spine surgery experienced more intense fluctuation in functional capacity than those who had other noncardiac surgeries. The former usually encountered little confusion in ordinary life but suffered severe disability after surgery, whereas the latter often experienced oppositely. In our study, only 94 (14.4%) of the participants who underwent orthopaedic and spine surgeries and developed disability were underpowered to build a logistic model. Accordingly, subgroup analysis was performed on the basis of the complementary population in compromise.

Research on the kinetics of troponin T in MINS is limited. Evidence from acute myocardial infarction may shed some light on the explanation of our conclusion. When an ischaemic attack leads to imbalance between oxygen supply and demand, lactic acid accumulation in cardiomyocytes results in acidification of the intracellular environment (∼22–37 h after MI), which activates proteolytic and apoptotic enzymes. Troponin T is broken down into smaller fragments, which could pass through the intact cell membrane, and some of them with certain part are detected as whole troponin T by current commercial troponin T assay [17,18]. A study showed that the degree of proteolytic degradation increased with increasing severity of myocardial injury, as estimated by the total troponin level [19]. On the basis of the above findings, we speculated that patients with MINS 24 h after surgery have higher ratio of proteolytic fragments keeping certain stable part, which reflects more deteriorated intracellular environment and worse prognosis.

Our finding is consistent with that of a subanalysis of the ENIGMA-II trial, in which troponin elevation, excluding extracardiac aetiology, with or without any postoperative adverse events, was associated with worse presence of Katz activities of daily living inventory, reflecting overall performance in fundamental physical activity [20]. Disability in this research, defined by a more comprehensive scale, was measured using the telephone-based version of WHODAS 2.0, containing six important domains, namely, cognition, mobility, self-care, interpersonal relationships, work and household roles and participation in society, which has been evaluated in diverse surgical cohort with different degrees of comorbidity [14]. Meanwhile, elevated troponin levels are independently associated with self-reported health status (assessed by EuroQol five-dimensional questionnaire, EQ-5D) 1 year after surgery [21]. Whilst both EQ-5D and WHODAS 2.0 are assessments recommended by StEP-COMPAC working group to evaluate patient-centred outcomes, EQ-5D is a measure of health-related quality of life, and the latter is a measure of functional outcome; WHODAS 2.0 was classified under the same domain with EQ-5D for the entire Delphi consensus process until the final stage [12]. Although functional capacity certainly affects quality of life, items in the two measurements are overlapping in some degree. Hence, troponin may be a potential biomarker for predicting recovery quality after a long time, which also helps to answer the questions on patient care and facilitates recovery plans after discharge.

Despite the varying conclusions drawn from a multicentre cohort study adopting the same disability assessment, patients with troponin exceeding the 99th percentile reported similar disability scores at 6 months [22]. The differences in methods and definitions between the two studies should be recognised. The first difference was the threshold of troponin elevation, which was defined as troponin exceeding the 99th percentile of the used assay or an increase of 20% of the preoperative troponin in the multicentre cohort study and a change of at least 5 ng/L or a postoperative troponin T level of at least 65 ng/L in the current study. The second difference lies in inclusion criteria. Participants of various age groups were enrolled across centres (i.e. participants aged ≥50 or 60 year, from different hospitals, were enrolled) in that multicentre study. By contrast, our cohort enrolled patients aged ≥45 year with cardiovascular diseases or cardiovascular risk factors scheduled for major noncardiac surgery, alike to the subanalysis of the ENIGMA-II trial. Peak troponin level was associated with poor long-term prognosis in a dose-dependent manner. However, in this setting, the peak troponin levels in the two studies were difficult to compare directly or indirectly. The negative implications of cardiovascular diseases or cardiovascular risk factors on quality of life and survival were well known. Population difference may be the key to interpreting the discrepancy, with heterogeneity in the threshold of troponin elevation.

Findings generated from our data provide some implications to clinical practice and further research. Firstly, our data revealed a link between MINS occurring after 24 h and long-term functional capacity, beyond the prognostic capability of major adverse cardiovascular events or death following surgery [5]. The misallocation of health care-related resources is a fundamental global issue, in which the high concentration of medical services in economically developed or capital cities stimulates remote health care-seeking behaviour, namely, patients are willing to pay a high price to access high-quality perioperative care [23,24]. In actuality, after surgery, patients usually recover shortly during hospitalisation and then experience a long rehabilitation of functional capacity in their home city. Therefore, a suitable expectation of rehabilitation and follow-up plan at the time of discharge could provide patients accurate information, relieve patients’ anxiety and avoid unnecessary follow-up. Our observations suggested that the occurrence time of MINS is an alternative reference to judge functional capacity and facilitate follow-up plan.

Secondly, this research supports current practice guidelines in recommending routine troponin T surveillance for high-risk individuals in perioperative periods [6–8], especially 24 h postoperatively. Troponin T assay is the only way to detect MINS; however, the implementation of troponin T surveillance is unsatisfactory, even after perioperative troponin monitoring endorsed by relevant professional societies [25]. Some clinicians argued that troponin testing before surgery is costly and useless, not to mention surveillance in perioperative periods, considering the lack of clinically recognised management strategies [26]. The recommendation that routine troponin T surveillance is economically acceptable and benefits patients at increased cardiovascular risk was proved in Canadian patients who participated in the VISION study [27]. Previous study generated from PREVENGE-CB revealed that preoperative troponin T improve the prediction for cardiovascular events within 30 days after noncardiac surgery and a recent study also revealed that troponin increase facilitates prognostic stratification for patients with non-ST-segment-elevation myocardial infarction after percutaneous coronary intervention [11,28]. Another exploration suggested that elevated troponin within 24 h of elective noncardiac surgery is associated with the development of noncardiac complications and that troponin monitoring potentially offers a real-time assessment tool to assign postoperative care [10]. Our observation expands that even slight increments in troponin 24 h postoperatively are prognostically relevant and that routine detection will help flag patients with poor functional capacity in early postoperative periods, although most MINS were diagnosed on the day of surgery or the first postoperative day [5]. Thirdly, our conclusion reinforces the significance of understanding pathophysiology of MINS and mechanisms underlying troponin T release in perioperative periods and appeals to complement patient-centred outcomes in further study.

To the best of our knowledge, this is the first study to explore the association between different occurrence time of MINS and disability at 180 days after major noncardiac surgery. In this cohort, a high satisfactory rate of troponin T surveillance completeness of >98% was achieved, before and after surgery, and telephone follow-up response rate (>92%) was also high at 180 days after surgery. However, the observational design limits bias control, this cohort did not involve information on the time frame or occurrence time of other postoperative adverse events and type of cancer, some other pathophysiological processes may cause severe damage to long-term functional capacity, malignant tumor and subsequent treatment undoubtly worsen quality of life. This cohort only measured troponin T obligatorily on the first second days after surgery, the subsequent postoperative troponin T was tested based on clinical necessity, which may result in the omission of MINS after 24 h. Besides, the generalisability of our findings may be limited to population with increased cardiovascular risk undergoing major surgery, disability associated with MINS occurring in lower risk patients and patients undergoing minor surgery still unknown. Furthermore, the exposure grouping stratified according to the kinetics of troponin T in acute myocardial infarction may be controversial, although the aetiologies of MINS and acute myocardial infarction are both related to myocardial ischemia.

## Conclusion

In summary, MINS occurring after 24 h deteriorates the long-term functional capacity in high-cardiovascular-risk population, regardless of whether it is accompanied by other complications or not. A proactive perioperative troponin T surveillance strategy for high-risk population, especially measurement 24 h postoperatively, provides an objective reference to stratify recovery of long-term functional capacity and facilitate follow-up schedule.

## Supplementary Material

Supplementary material.docx

## Data Availability

The datasets generated and analysed during the current study are not publicly available because of the Nanfang Hospital regulations but are available from the corresponding author Ke-Xuan Liu on reasonable request.

## Abbreviations

AKI: Acute kidney injury
BMI: Body mass index
eGFR: Estimated glomerular filtration rate
