# Supplementary material for: Association between occurrence time of myocardial injury after noncardiac surgery and long-term functional capacity: a secondary analysis of a prospective study
Source: Ann Med. 2025 Sep 2;57(1):2552936. doi: 10.1080/07853890.2025.2552936 (PMC12406311; doi:10.1080/07853890.2025.2552936)
Supplement: Supplementary material.docx [file IANN_A_2552936_SM2926.docx]

**Supplementary Material**

In the present analysis, we merged some comorbidities or complications according to pathophysiology or anatomy. Specifically, infection includes surgical site infection, pneumonia, sepsis and unknown infection. Pulmonary complications include respiratory failure, atelectasis and pulmonary aspiration. Intestinal complications include postoperative ileus, anastomosis leak and intestinal bleeding. Detailed definitions and diagnostic criteria are as follow:

(1) Myocardial infarction, a diagnosis requiring both MINS and at least one of the following:

a. ischemic signs or symptoms (i.e., chest, arm, neck, or jaw discomfort; shortness of breath; or pulmonary edema);

b. new or presumed new electrocardiography (ECG) changes indicative of ischemia (i.e., ST segment elevation ≥2 mm in leads V1, V2, or V3 or ≥1 mm in the other leads; ST segment depression ≥ 1 mm; or symmetric inversion of T waves ≥ 1 mm) in at least two contiguous leads;

c. new left bundle branch block (LBBB);

d. development of pathologic Q waves in any two contiguous leads that were ≥30 milliseconds;

e. new cardiac wall motion abnormality on echocardiography or new fixed defect on radionuclide imaging; f. identification of intracoronary thrombus on angiography or autopsy.

(2) Coronary revascularization, defined as percutaneous coronary intervention or coronary artery bypass graft surgery.

(3) New atrial fibrillation, defined as new onset of atrial fibrillation or flutter lasting at least 30 s or for the duration of the ECG recording (if <30 s).

(4) Cardiac arrest, including either documented or presumed ventricular fibrillation, sustained ventricular tachycardia, asystole, or pulseless electrical activity requiring cardiopulmonary resuscitation, pharmacological therapy, or cardiac defibrillation.

(5) Stroke, defined as new cerebral infarction or hemorrhage on CT or magnetic resonance imaging (MRI) scan; or new focal neurological deficit, suspected to vascular in origin, with signs/symptoms lasting ≥24hours.

(6) Pulmonary embolism, a diagnosis requiring any one of the following:

a. a high probability ventilation/perfusion lung scan;

b. an intraluminal filling defect of segmental or larger artery on a helical computed tomography (CT) scan;

c. an intraluminal filling defect on pulmonary angiography;

d. a positive diagnostic test for deep venous thrombosis (e.g., positive compression ultrasound) and one of the following: non-diagnostic (i.e., low or intermediate probability) ventilation/perfusion lung scan, or a non-diagnostic (i.e., subsegmental defects or technically inadequate study) helical CT scan.

(7) Cardiovascular death, defined as death with a possible cardiovascular cause, including deaths after myocardial infarction, coronary revascularization, arrythmia, cardiac arrest, heart failure, pulmonary embolism and stroke, and deaths of unknown cause.

(8) Surgical site infection (SSI), requiring surgeon/treating physician diagnosis of SSI, including superficial, deep and organ/space SSI, within 30 days after surgery.

(9) Sepsis, defined as the presence of an infection and an increase in Sequential Organ Failure Assessment (SOFA) score of 2 or more.

(10) Pneumonia, a diagnosis requiring any one of the following:

a. rales or dullness to percussion on physical examination of the chest and any of the following:

• new onset of purulent sputum or change in character of sputum;

• isolation of organism from blood culture;

• isolation of pathogen from specimen obtained by transtracheal aspirate, bronchial brushing, or biopsy;

b. chest radiography showing new or progressive infiltrate, consolidation, cavitation, or pleural effusion,

and any of the following:

• new onset of purulent sputum or change in character of sputum;

• isolation of organism from blood culture;

• isolation of pathogen from specimen obtained by transtracheal aspirate, bronchial brushing, or biopsy;

• isolation of virus or detection of viral antigen in respiratory secretions;

• diagnostic single antibody titer (IgM) or fourfold.

(11) Respiratory failure, a diagnosis based on the Berlin definition for acute respiratory distress syndrome, or the need for tracheal re-intubation and mechanical ventilation after extubation, or the need for mechanical ventilation for more than 24 h after surgery.

(12) Atelectasis, defined as active collapse of lungs from deficiency of inspiratory power. Identified on chest X-ray as a loss of lung volume, or on a CT scan – pixels with attenuation values of -100 to +100 hounsfield unit (HU) that occur in the most dependent parts of the lungs.

(13) Pulmonary aspiration, a diagnosis requiring clear clinical history and radiological evidence.

(14) Acute kidney injury, defined using the Kidney Disease Improving Global Outcomes (KDIGO) creatinine criteria, including stage 1, increase in SCr ≥ 0.3 mg/dL (in 48 hours) or 1.5 to 1.9 multiplied by baseline (in 7 days); stage 2, 2.0 to 2.9 multiplied by baseline SCr; and stage 3: 3.0 or more multiplied by baseline, increase in SCr ≥ 4.0 mg/dL, or beginning of renal replacement therapy regardless of a previous KDIGO stage.

(15) Postoperative ileus, defined as any nasogastric tube use or NPO (nil per os) status on postoperative day 4 or later.

(16) Anastomosis leak, requiring surgeon/treating physician diagnosis of anastomosis leak.

(17) Major bleeding, defined as bleeding that results in a drop in hemoglobin to <70 g/L, transfusion of ≥1 unit of packed red blood cells, or death.

|  | **Item No** | **Recommendation** |  | **Reported on page #** |  |
| --- | --- | --- | --- | --- | --- |
| **Title and abstract** | 1 | (*a*) Indicate the study’s design with a commonly used term in the title or the abstract |  | Page 1 |  |
|  |  | (*b*) Provide in the abstract an informative and balanced summary of what was done and what was found |  | Pages 2-3 |  |
| **Introduction** | | |  |  |  |
| Background/rationale | 2 | Explain the scientific background and rationale for the investigation being reported |  | Pages 4-5 |  |
| Objectives | 3 | State specific objectives, including any prespecified hypotheses |  | Page 5 |  |
| **Methods** | | |  |  |  |
| Study design | 4 | Present key elements of study design early in the paper |  | Page 5 |  |
| Setting | 5 | Describe the setting, locations, and relevant dates, including periods of recruitment, exposure, follow-up, and data collection |  | Pages 5-7 |  |
| Participants | 6 | (*a*) Give the eligibility criteria, and the sources and methods of selection of participants. Describe methods of follow-up |  | Pages 5-6 |  |
|  |  | (*b*) For matched studies, give matching criteria and number of exposed and unexposed |  | NA |  |
| Variables | 7 | Clearly define all outcomes, exposures, predictors, potential confounders, and effect modifiers. Give diagnostic criteria, if applicable |  | Pages 6-7 |  |
| Data sources/ measurement | 8 | For each variable of interest, give sources of data and details of methods of assessment (measurement). Describe comparability of assessment methods if there is more than one group |  | Pages 6-7 |  |
| Bias | 9 | Describe any efforts to address potential sources of bias |  | Pages 5-8 |  |
| Study size | 10 | Explain how the study size was arrived at |  | Page 5 |  |
| Quantitative variables | 11 | Explain how quantitative variables were handled in the analyses. If applicable, describe which groupings were chosen and why |  | Page 7 |  |
| Statistical methods | 12 | (*a*) Describe all statistical methods, including those used to control for confounding |  | Pages 7-8 |  |
|  |  | (*b*) Describe any methods used to examine subgroups and interactions |  | Pages 7-8 |  |
|  |  | (*c*) Explain how missing data were addressed |  | Page 5 |  |
|  |  | (*d*) If applicable, explain how loss to follow-up was addressed |  | Page 5 |  |
|  |  | (*e*) Describe any sensitivity analyses |  | Page 8 |  |
| **Results** | | |  |  |  |
| Participants | 13 | (a) Report numbers of individuals at each stage of study—eg numbers potentially eligible, examined for eligibility, confirmed eligible, included in the study, completing follow-up, and analysed |  | Page 8, Figure 1 |  |
|  |  | (b) Give reasons for non-participation at each stage |  | Figure 1 |  |
|  |  | (c) Consider use of a flow diagram |  | Figure 1 |  |
| Descriptive data | 14 | (a) Give characteristics of study participants (eg demographic, clinical, social) and information on exposures and potential confounders |  | Page 8, Table 1 |  |
|  |  | (b) Indicate number of participants with missing data for each variable of interest |  | Figure 1 |  |
|  |  | (c) Summarise follow-up time (eg, average and total amount) |  | Figure 1 |  |
| Outcome data | 15 | Report numbers of outcome events or summary measures over time |  | Page 9,  Table 1 |  |
| Main results | 16 | (*a*) Give unadjusted estimates and, if applicable, confounder-adjusted estimates and their precision (eg, 95% confidence interval). Make clear which confounders were adjusted for and why they were included |  | Page 9-10, Table 1 |  |
|  |  | (*b*) Report category boundaries when continuous variables were categorized |  | NA |  |
|  |  | (*c*) If relevant, consider translating estimates of relative risk into absolute risk for a meaningful time period |  | NA |  |
| Other analyses | 17 | Report other analyses done—eg analyses of subgroups and interactions, and sensitivity analyses |  | Page 10-11,  Tables 2-4,  Figure 2 |  |
| **Discussion** | | |  |  |  |
| Key results | 18 | Summarise key results with reference to study objectives |  | Page 11 |  |
| Limitations | 19 | Discuss limitations of the study, taking into account sources of potential bias or imprecision. Discuss both direction and magnitude of any potential bias |  | Page 16-17 |  |
| Interpretation | 20 | Give a cautious overall interpretation of results considering objectives, limitations, multiplicity of analyses, results from similar studies, and other relevant evidence |  | Pages 12-16 |  |
| Generalisability | 21 | Discuss the generalisability (external validity) of the study results |  | Page 16 |  |
| **Other information** | | |  |  |  |
| Funding | 22 | Give the source of funding and the role of the funders for the present study and, if applicable, for the original study on which the present article is based |  | Page 19 |  |

Table S1. STROBE Statement—Checklist of items that should be included in reports of cohort studies

|  | All n=2469 | Before weighting | | | | | | After IPW | | | | After OW | | | |  |
| --- | --- | --- | --- | --- | --- | --- | --- | --- | --- | --- | --- | --- | --- | --- | --- | --- |
|  |  | without MINS n=2208(89.4%) | MINS within 24h n=178(7.2%) | MINS after 24h n=83(3.4%) | *P*-value | | max SMD | | *P*-value | | max SMD | | *P*-value | | max SMD | |
| Age, yr | 68.0 (63.0,74.0) | 68.0 (63.0, 73.0) | 70.0 (65.0, 78.0) | 72.0 (67.0, 79.0) | <0.001 | | 0.448 | | 0.773 | | 0.118 | | 0.923 | | 0.048 | |
| Female | 977(39.5) | 887 (40.2) | 54 (30.3) | 36 (43.4) | 0.028 | | 0.273 | | 0.362 | | 0.199 | | 0.806 | | 0.0603 | |
| Body mass index, kg/m^2^ | 23.1 (20.8,25.5) | 23.2 (20.8, 25.5) | 22.4 (20.0, 24.7) | 22.2 (20.5, 25.6) | 0.018 | | 0.211 | | 0.716 | | 0.157 | | 0.708 | | 0.106 | |
| Smoke |  |  |  |  | 0.003 | | 0.264 | | 0.129 | | 0.346 | | 0.993 | | 0.037 | |
| Never | 1562(63.3) | 1405 (63.6) | 100 (56.2) | 57 (68.7) |  | |  | |  | |  | |  | |  | |
| Former | 270(10.9) | 225 (10.2) | 33 (18.5) | 12 (14.5) |  | |  | |  | |  | |  | |  | |
| Current | 637(25.8) | 578 (26.2) | 45 (25.3) | 14 (16.9) |  | |  | |  | |  | |  | |  | |
| Cardiovascular comorbidities | 1624(65.8) | 1446 (65.5) | 122 (68.5) | 56 (67.5) | 0.674 | | 0.065 | | 0.484 | | 0.168 | | 0.694 | | 0.092 | |
| Chronic obstructive pulmonary disease | 86(3.5) | 73 (3.3) | 10 (5.6) | 3 (3.6) | 0.269 | | 0.112 | | 0.839 | | 0.046 | | 0.893 | | 0.038 | |
| Diabetes mellitus |  |  |  |  | 0.003 | | 0.354 | | 0.558 | | 0.241 | | 0.963 | | 0.061 | |
| No | 1713(69.4) | 1540 (69.7) | 109 (61.2) | 64 (77.1) |  | |  | |  | |  | |  | |  | |
| Diabetes mellitus on  oral drug | 525(21.3) | 475 (21.5) | 40 (22.5) | 10 (12.0) |  | |  | |  | |  | |  | |  | |
| Diabetes mellitus on insulin | 231(9.4) | 193 (8.7) | 29 (16.3) | 9 (10.8) |  | |  | |  | |  | |  | |  | |
| Liver | 607(24.6) | 554 (25.1) | 36 (20.2) | 17 (20.5) | 0.237 | | 0.116 | | 0.661 | | 0.109 | | 0.903 | | 0.039 | |
| Cancer | 1467(59.4) | 1317 (59.6) | 103 (57.9) | 47 (56.6) | 0.781 | | 0.061 | | 0.564 | | 0.187 | | 0.913 | | 0.038 | |
| eGFR, mL/min | 84.4 (69.5,92.7) | 85.3 (70.9, 93.1) | 74.1 (54.2, 86.8) | 72.7 (53.6, 87.0) | <0.001 | | 0.578 | | 0.824 | | 0.071 | | 0.957 | | 0.0304 | |
| Albumin, g/L | 38.0(35.0,40.6) | 38.1 (35.2, 40.7) | 36.8 (33.2, 39.6) | 36.5 (33.6, 38.5) | <0.001 | | 0.374 | | 0.961 | | 0.05 | | 0.861 | | 0.073 | |
| Hemoglobin, g/L | 123.0 (107.0,135.0) | 124.0  (108.0, 136.0) | 117.0  (98.3, 131.0) | 116.0  (102.0, 131.0) | <0.001 | | 0.289 | | 0.899 | | 0.097 | | 0.912 | | 0.060 | |
| Minimally invasive surgery | 1470(59.5) | 1337 (60.6) | 95 (53.4) | 38 (45.8) | 0.006 | | 0.299 | | 0.576 | | 0.143 | | 0.931 | | 0.033 | |
| Blood transfusion during surgery | 275(11.1) | 218 (9.9) | 43 (24.2) | 14 (16.9) | <0.001 | | 0.387 | | 0.686 | | 0.078 | | 0.867 | | 0.042 | |
| Type of surgery |  |  |  |  | 0.081 | | 0.296 | | 0.897 | | 0.236 | | >0.999 | | 0.079 | |
| Foregut or hepatopancreatobiliary | 566(22.9) | 508 (23.0) | 45 (25.3) | 13 (15.7) |  | |  | |  | |  | |  | |  | |
| Intestinal | 480(19.4) | 438 (19.8) | 26 (14.6) | 16 (19.3) |  | |  | |  | |  | |  | |  | |
| Orthopedic | 434(17.6) | 371 (16.8) | 42 (23.6) | 21 (25.3) |  | |  | |  | |  | |  | |  | |
| Thoracic(nonesophageal) | 371(15.0) | 333 (15.1) | 23 (12.9) | 15 (18.1) |  | |  | |  | |  | |  | |  | |
| Urological | 250(10.1) | 217 (9.8) | 24 (13.5) | 9 (10.8) |  | |  | |  | |  | |  | |  | |
| Spine | 219(8.9) | 203 (9.2) | 11 (6.2) | 5 (6.0) |  | |  | |  | |  | |  | |  | |
| Other* | 149(6.0) | 138 (6.2) | 7 (3.9) | 4 (4.8) |  | |  | |  | |  | |  | |  | |
| Type of anesthesia |  |  |  |  | 0.004 | | 0.239 | | 0.803 | | 0.135 | | 0.992 | | 0.039 | |
| Regional anesthesia alone | 269(10.9) | 223 (10.1) | 31 (17.4) | 15 (18.1) |  | |  | |  | |  | |  | |  | |
| General plus regional anesthesia | 54(2.2) | 47 (2.1) | 6 (3.4) | 1 (1.2) |  | |  | |  | |  | |  | |  | |
| General anesthesia alone | 2146(86.9) | 1938 (87.8) | 141 (79.2) | 67 (80.7) |  | |  | |  | |  | |  | |  | |
| Cardiovascular complications | 98(4.0) | 60 (2.7) | 27 (15.2) | 11 (13.3) | <0.001 | | 0.447 | | 0.731 | | 0.054 | | 0.744 | | 0.086 | |
| AKI | 180(7.3) | 134 (6.1) | 33 (18.5) | 13 (15.7) | <0.001 | | 0.387 | | 0.302 | | 0.186 | | 0.988 | | 0.014 | |
| Infection | 203(8.2) | 157 (7.1) | 32 (18.0) | 14 (16.9) | <0.001 | | 0.333 | | 0.533 | | 0.156 | | 0.726 | | 0.089 | |
| Pulmonary complicatoins | 18(0.7) | 13 (0.6) | 3 (1.7) | 2 (2.4) | 0.048 | | 0.15 | | 0.276 | | 0.065 | | 0.903 | | 0.036 | |
| Intestinal complications | 69(2.8) | 65 (2.9) | 4 (2.2) | 0 (0.0) | 0.251 | | 0.246 | | 0.414 | | 0.283 | | 0.243 | | <0.001 | |
| Major bleeding | 81(3.3) | 59 (2.7) | 16 (9.0) | 6 (7.2) | <0.001 | | 0.272 | | 0.666 | | 0.065 | | 0.948 | | 0.330 | |
| Values in parentheses are percentages or interquartile ranges | | |  |  |  |  | |  | |  | |  | |  | |  |
| *Included gynecologic (81, 54.3%), head and neck (47, 31.5%), and vascular (21, 14.1%) surgery | | | |  |  |  | |  | |  | |  | |  | |  |
| max, maxium |  |  |  |  |  |  | |  | |  | |  | |  | |  |

Table S2. Characteristics of participants stratified by MINS

| Variable | VIF |
| --- | --- |
| Age, yr | 1.429 |
| Female | 1.638 |
| Body mass index, kg/m^2^ | 1.53 |
| Smoke | 1.281 |
| Cardiovascular comorbidities | 1.446 |
| Chronic obstructive pulmonary disease | 1.647 |
| Diabetes mellitus | 1.165 |
| Liver | 1.327 |
| Cancer | 1.147 |
| eGFR, mL/min | 1.029 |
| Albumin, g/L | 1.062 |
| Hemoglobin, g/L | 1.051 |
| Minimally invasive surgery | 1.696 |
| Blood transfusion during surgery | 1.305 |
| Type of surgery | 1.43 |
| Type of anesthesia | 1.686 |
| MINS | 1.106 |
| Cardiovascular complications | 1.174 |
| AKI | 1.062 |
| Infection | 1.195 |
| Pulmonary complicatoins | 1.125 |
| Intestinal complications | 1.073 |
| Major bleeding | 1.062 |

Table S3. Variance Inflation Factor (VIF) results for multicollinearity assessment
